# *SCD* rs41290540 single‐nucleotide polymorphism modifies miR‐498 binding and is associated with a decreased risk of coronary artery disease

**DOI:** 10.1002/mgg3.1136

**Published:** 2020-01-21

**Authors:** Zhou Liu, Xiaojian Yin, Hui Mai, Guangning Li, Zhijun Lin, Wanxin Jie, Kanglan Li, Haihong Zhou, Shouchao Wei, Li Hu, Wanjuan Peng, Jiajing Lin, Feng Yao, Hua Tao, Xing‐dong Xiong, Keshen Li

**Affiliations:** ^1^ Department of Neurology Guangdong Key Laboratory of Age‐Related Cardiac and Cerebral Diseases Institute of Neurology Affiliated Hospital of Guangdong Medical University Zhanjiang China; ^2^ Department of Neurology Huadu District People’s Hospital Southern Medical University Guangzhou China; ^3^ Cardiovascular Medicine Center Affiliated Hospital of Guangdong Medical University Zhanjiang China; ^4^ Institute of Aging Research Guangdong Medical University Dongguan China; ^5^ Stroke Center Neurology & Neurosurgery Division Clinical Neuroscience Institute & The First Affiliated Hospital Jinan University Guangzhou China

**Keywords:** 3′‐untranslated region, coronary artery disease, microRNA, single‐nucleotide polymorphism, stearoyl‐CoA desaturase

## Abstract

**Background:**

Atherosclerosis is the primary cause of coronary artery disease (CAD), and stearoyl‐CoA desaturase (SCD) is associated with atherosclerosis. However, the associations between variants of *SCD* and CAD have not yet been decided.

**Methods:**

This study analyzed *SCD* rs41290540 single‐nucleotide polymorphism (SNP) in the 3′‐untranslated region for an association with a risk of CAD among the Chinese Han population. CAD patients and controls were genotyped for SNP rs41290540 in *SCD* by SNaPshot. The binding affinity of miR‐498 to rs41290540 was determined by a luciferase assay, and SCD expression was assessed using Western blot.

**Results:**

A total of 969 CAD patients and 1,095 control subjects were involved in this study. The *SCD* rs41290540CC genotype is associated with a decreased risk of CAD compared with the AA genotype. Furthermore, the CC genotype is associated with lower serum total cholesterol (TC). Western blot analysis demonstrated that miR‐498 suppressed the expression of SCD. A luciferase assay confirmed that rs41290540 A>C variation in the *SCD* 3′UTR inhibits miR‐498 binding.

**Conclusion:**

This study demonstrates that the *SCD* rs41290540 may be associated with a decreased risk of CAD, lower serum TC, and decreased miR‐498 binding.

## Graphical abstract

We find that the stearoyl‐CoA desaturase rs41290540 may reduce the risk of coronary artery disease. Furthermore, we prove that rs41290540 works by reducing miR‐498 binding.

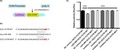

## 1 INTRODUCTION

Coronary artery disease (CAD) is one of the main causes of death worldwide (Lopez, Mathers, Ezzati, Jamison, & Murray, 2006) and is a disease of complicated etiopathogenesis involving interactions between multiple genetic factors (Sayols‐Baixeras, Lluis‐Ganella, Lucas, & Elosua, 2014) and between genetic and environmental factors. In addition, hypertension, diabetes, hyperlipoidemia, and metabolic syndrome are risk factors for CAD (Lloyd‐Jones et al., 2010; Wilson, D'Agostino, Parise, Sullivan, & Meigs, 2005). These factors promote atherosclerotic plaque formation and therefore increase the risk of CAD.

Atherosclerosis, the primary cause of CAD, is widely acknowledged to be due to inflammation. Saturated fatty acids (SFA) represent potential contributors to atherosclerosis (Krogmann et al., 2011; Simon et al., 1995; Yamagishi et al., 2013). A proposed mechanism could be the induction of inflammatory cytokines in arterial endothelial cells. Palmitate, the most plentiful SFA in human plasma, triggers the expression of IL1A, IL6, IL8, CXCL2, CXCL3, CCL20, SPP1, and CEBPB (Krogmann et al., 2011). SFA may induce atherosclerosis by triggering inflammation and by inducting endothelial cell apoptosis (Artwohl, Roden, Waldhausl, Freudenthaler, & Baumgartner‐Parzer, 2004; Li, Gonzalez, et al., 2013). Moreover, SFAs rise total cholesterol (TC) and low‐density lipoprotein cholesterol (LDL‐C) (Mattson & Grundy, 1985; Mensink & Katan, 1992), widely used CAD markers. However, monounsaturated fatty acids (MUFAs), desaturated fatty acids produced from SFA through catalysis by stearoyl‐CoA desaturase (SCD), can reduce inflammation, apoptosis (Harvey et al., 2010; Magdalon et al., 2012; Staiger et al., 2006), and high TC and LDL‐C associated with SFA (Mattson & Grundy, 1985; Mensink & Katan, 1992). MUFA plays a beneficial role in CAD (Matsumoto, Matthan, Lichtenstein, Gaziano, & Djousse, 2013). Therefore, enhancing the activity of SCD may be a strategy to reduce the risk of CAD. Recent studies have demonstrated that macrophage SCD attains an antiatherogenic result by boosting reverse cholesterol transport to high‐density lipoprotein (HDL) (Nakaya et al., 2013) and protects against lipotoxicity (Peter et al., 2008). Moreover, mice in which SCD is inhibited and SCD‐knockdown mice have increased atherosclerosis (Brown et al., 2008; MacDonald et al., 2009).

Human *SCD* (OMIM# 604031) (also known as *SCD1*) is located on chromosome 10q24.31. Variations in the *SCD* in humans alter enzyme activity (Stryjecki et al., 2012) and might be involved in individual susceptibility to obesity (Martin‐Nunez et al., 2013), C‐reactive protein (CRP) levels (Stryjecki et al., 2012), metabolic syndrome (Gong et al., 2011), and insulin sensitivity (Warensjo et al., 2007). However, the associations between variants of *SCD* and CAD have not yet been decided. We therefore took on a case‐control study to ascertain whether the rs41290540 single‐nucleotide polymorphism (SNP) of *SCD* is associated with CAD susceptibility and, if so, to analyze its underlying mechanism.

## 2 METHODS

### 2.1 Study population

Our study included 969 unrelated individuals of Han Chinese with CAD (600 from the Dongguan People's Hospital, Guangdong; 369 from the Affiliated Hospital of Guangdong Medical University) and 1,095 controls (684 from the Dongguan People's Hospital, Guangdong; 411 from the Affiliated Hospital of Guangdong Medical University).

The definition of CAD was significant coronary stenosis (≥50%) in no less than one third of main coronary arteries or their major branches (branch diameter ≥2 mm). The controls were determined to be free of CAD according to medical history, questionnaires, electrocardiography, and clinical examination.

Diagnosis of hypertension was established if patients were taking antihypertensive medication, if the mean of three resting measurements of systolic blood pressure was above 140 mmHg, or diastolic blood pressure (DBP) was above 90 mmHg. Diabetes mellitus was diagnosed by a fasting blood glucose (FBG) above 7.0 mmol/L or by use of antidiabetic drug therapies. Hyperlipidemia was diagnosed by a TC ≥5.72 mmol/L and/or triglyceride ≥1.7 mmol/L. The research was approved by the Ethical Committees of the Dongguan People's Hospital and the Ethical Committees of the Affiliated Hospital of Guangdong Medical University. Written informed consent conforming to the tenets of the Declaration of Helsinki (1983 Revision).

### 2.2 DNA isolation and genotyping

DNA isolation and genotyping were conducted as stated in our previous reports (Li, Liao, et al., 2013). Genomic DNA was extracted from venous blood using the EZ‐10 Spin Column Whole Blood Genomic DNA Isolation Kit (Sangon Biotech), following instruction of the manufacturer.

The genotypes were determined by SNaPshot Multiplex Kit (Applied Biosystems Co., Ltd.). SNaPshot responses were carried out in a 10 μL total volume that contains 5‐μL SNaPshot Multiplex Kit (ABI), 1‐μL primer mix, 2‐μL water, and 2‐μL templates comprising the multiplex PCR productions from the different genes. The SNaPshot procedure consisted of (a) initial denaturation of 1 min at 96°C, (b) denaturation of 10 s at 96°C, (c) annealing of 5 s at 52°C, and (d) extension of 30 s at 60°C for a total of 28 cycles. Amplified samples were stored at 4°C. Extension products were purified by incubation of 1 hr with 1 U of shrimp alkaline phosphatase (Takara) at 37°C and enzyme inactivation of 15 min at 75°C. For capillary electrophoresis, 0.5 μL of purified products were mixed with 9 μL of Hi‐Di Formamide and 0.5‐μL GeneScan 120 Liz Size Standard. Samples were incubated at 95°C for 5 min and then run on an ABI 3130xl Genetic Analyzer. The outcomes were analyzed by GeneMapper 4.0 (Applied Biosystems Co., Ltd.).

### 2.3 Cell lines and cell culture

Human aortic smooth muscle cells (HASMCs) (Shanghai Institute of Cell Biology, China) and human embryonic kidney (HEK) 293T cells (Shanghai Institute of Cell Biology) were cultured in 24‐well plates with 0.5 ml of DMEM/F‐12 containing 10% fetal bovine serum (FBS), 100 U/mL penicillin, and 100 μg/mL streptomycin. The cells were cultured in a 5% CO_2_ humidified incubator at 37°C. Transfection was carried out while the cells achieved a confluence of 80%.

### 2.4 Prediction of miRNAs binding to the SNP

TargetScan Version 7.1 (http://www.targetscan.org/vert_71/) was used to predict potential miRNAs with binding sites in SNPs in the *SCD* 3′UTR.

### 2.5 Dual‐luciferase assay

A full‐length of the *SCD* 3′UTR containing rs41290540 (A/C, wild type/mutant type) (Hanbio Biotechnology Co., Ltd.) was synthesized in vitro and cloned into the downstream region of the pMIR‐REPORT miRNA Expression Reporter Vector System (Hanbio Biotechnology Co., Ltd.) using the XhoI and NotI enzymes (Fermentas). HEK 293T cells were co‐transfected with miR‐498 Mimic/Negative.Control (N.C)/inhibitor/inhibitor N.C (Hanbio Biotechnology Co., Ltd.) and wild‐type *SCD* 3′UTR or mutant 3′UTR. Following transfection for 48 hr, cells were harvested, and luciferase activity was measured with a GloMax 20/20 luminometer (Promega Corporation) based on the standard protocol of the Dual Luciferase Reporter Gene Assay kit (Promega Dual‐Luciferase system). The intensity of Renilla luciferase was used as the control and the fluorescence intensity in different groups was analyzed.

### 2.6 Western blot

For transfection of miR‐498 Mimic/Negative.Control (N.C)/inhibitor/inhibitor N.C (Hanbio Technology Co., Ltd.), HASMCs were cultured in antibiotic‐free H‐DMEM medium with 10% FBS; 1.25‐µl miR‐498 Mimic/Negative.Control (N.C)/inhibitor/inhibitor N.C and 1‐µl Lipofectamine^®^ 2000 (Invitrogen; Thermo Fisher Scientific, Inc.) were added to tubes containing 50‐µl Opti‐MEM medium (Invitrogen; Thermo Fisher Scientific, Inc.). Cells were harvested 48 hr following transfection, and proteins were extracted with RIPA buffer containing phenylmethanesulfonyl fluoride (Beyotime). Equivalent proteins were separated by 12.5% sodium dodecyl sulfate polyacrylamide gel electrophoresis and blotted onto polyvinylidene fluoride membranes. Primary polyclonal antibodies of SCD were obtained from ABCAM, UK and GAPDH was obtained from Santa Cruz, USA. The secondary antibodies were HRP‐linked anti‐rabbit from Santa Cruz Biotechnology. The blots were developed by ECL reagent (Millipore). Equivalent proteins were loaded into each lane and loaded amounts were confirmed using GAPDH antibody.

### 2.7 Statistical analyses

Chi‐square tests, *t* tests, and one‐way ANOVA tests were executed using SPSS 19.0 software (IBM). Codominant, dominant, and recessive genetic models of inheritance were selected to value the connection between CAD and the SNP by SNPStats (http://bioinfo.iconcologia.net/SNPstats) (Solé, Guinó, Valls, Iniesta, & Moreno, 2006). Multiple logistic regression models were used to calculate the odds ratio (OR), 95% confidence interval (CI), and *p* values in three genetic models adjusted by age, sex, hypertension, diabetes mellitus, and hyperlipidemia. Significance level of 0.05 was applied. The statistical power was also calculated using the Quanto (Version 1.2.4, University of Southern California).

## 3 RESULTS

### 3.1 Baseline characteristics

Our study consecutively recruited 2064 participants, 969 CAD patients, and 1,095 controls patients. The baseline characteristics of patients with CAD and control patients are summed up in Table 1. The mean age of the CAD and control group was 65.5 ± 12.0 and 59.8 ± 11.9, respectively. Compared with the control group, more subjects in the CAD group had hypertension, diabetes, and dyslipidemia, and had larger average fasting plasma glucose, triglycerides, and LDL cholesterol, and had a lower average HDL cholesterol. TC in the CAD patients was considerably lower than in the control group, possibly resulting from treatment of CAD patients with hyperlipidemia.

**Table 1:** The characteristics of subjects in the CAD and control groups

Variables	CAD (*n* = 969)	Control (*n* = 1,095)	*p* value
Age (years)	65.5 ± 12.0	59.8 ± 11.9	<.001
Male/female	637/332	673/422	.044
Fasting glucose (mmol/L)	6.6 ± 1.9	5.7 ± 1.7	<.001
Triglycerides (mmol/L)	1.9 ± 1.1	1.5 ± 1.0	<.001
Total cholesterol (mg/dl)	4.7 ± 1.3	4.9 ± 1.2	<.001
HDL (mmol/L)	1.2 ± 0.4	1.5 ± 0.6	<.001
LDL (mmol/L)	2.9 ± 1.0	2.8 ± 1.0	.013
Hypertension, *n*	590	345	<.001
Diabetes, *n*	381	141	<.001
Hyperlipidemia, *n*	462	289	<.001

Note

Continuous data are expressed as the means ± *SD*.

Abbreviations: CAD, Coronary artery disease; HDL, high‐density lipoprotein; LDL, low‐density lipoprotein.

### 3.2 Rs41290540 and the risk of CAD

Deviation from the Hardy–Weinberg equilibrium was not significant (data not shown). The genotype and allele frequency of rs41290540 in all subjects are shown in Table 2. The frequency of the CC genotype of rs41290540 was significantly lower in the CAD group than in the control group (2.4% vs. 1%). After adjustments for age, sex, hypertension, diabetes mellitus, and hyperlipidemia, the CC genotype of rs41290540 was identified as a protective factor against CAD in both the codominant and recessive models (OR = 0.32, 0.31, 95% CI = 0.14–0.72, 0.14–0.70, *p* = .009,0.0032, respectively). Our sample size provided sufficient statistical power (>99% at Type I error rate of 0.05).

**Table 2:** Frequency of rs41290540 genotype in CAD and control patients

Model	Genotype	CAD	Control	OR (95% CI)	*p*‐value	Adjusted *p*‐value
Codominant	A/A	681 (70.2%)	803 (73.3%)	1.00	.006	.009
	A/C	278 (28.7%)	267 (24.4%)	1.13 (0.91–1.41)		
	C/C	10 (1%)	25 (2.3%)	0.31 (0.14–0.71)		
Dominant	A/A	681 (70.2%)	803 (73.3%)	1.00	.85	.85
	A/C‐C/C	288 (29.8%)	292 (26.7%)	1.05 (0.84–1.30)		
Recessive	A/A‐A/C	959 (99%)	1,070 (97.7%)	1.00	.003	.009
	C/C	10 (1%)	25 (2.3%)	0.30 (0.14–0.68)		

Note

Adjusted for age, sex, hypertension, diabetes mellitus, and hyperlipidemia.

Abbreviation: CAD, coronary artery disease; CI, confidence interval; OR, odds ratio.

### 3.3 The association of *SCD* rs41290540 with demographic characteristics

The association of *SCD* rs41290540 with demographic features is displayed in Table 3. The analysis is stratified by hypertension, diabetes, and dyslipidemia. The risk associated with the variant genotypes and alleles was not detected.

**Table 3:** The relationship between baseline characteristics and the rs41290540 genotype and alleles among subjects in the case and control groups

Characteristics	CAD					Control				
	Genotype, *n*			Allele, *n*		Genotype, *n*			Allele, *n*	
	AA	AC	CC	A	C	AA	AC	CC	A	C
Hypertension										
Yes	415	166	9	996	184	251	82	12	584	106
No	266	112	1	644	114	552	185	13	1,289	211
Diabetes										
Yes	258	116	7	632	130	96	40	5	232	50
No	423	162	3	1,008	168	707	227	20	1,641	267
Dyslipidemia										
Yes	321	137	4	779	145	208	75	6	491	87
No	360	141	6	861	153	595	192	19	1,382	230

Abbreviation: CAD, coronary artery disease.

To determine the association of rs41290540 with clinical parameters, including fasting plasma glucose, TC, triglycerides, HDL cholesterol, and LDL cholesterol, we analyzed the association in the CAD group. The clinical parameters were not significantly different among the three genotypes (data not shown). Since the outcomes could be affected by taking hypoglycemic agents, lipid‐lowering drugs, and antihypertensive drugs, we analyzed the association in patients in the control group who did not have hypertension, diabetes, or hyperlipidemia. The results are shown in Table 4. All CC genotype carriers had lower clinical parameters; however, there was only a significant difference in TC in comparison with AA and AC genotype carriers in control subjects without hyperlipidemia (*p* < .05).

**Table 4:** Association of rs41290540 with blood lipid, fasting glucose, or blood pressure in the control group without hyperlipidemia, or diabetes mellitus, or hypertension

	A/A (*n* = 586)	A/C (*n* = 191)	C/C (*n* = 19)
TG (mol/L)	1.3 ± 0.8	1.3 ± 0.8	1.1 ± 0.3
TC (mg/dl)	4.8 ± 1.1*	4.9 ± 1.1*	4.3 ± 1.0
HDL (mol/L)	1.5 ± 0.4	1.6 ± 1.1	1.3 ± 0.4
LDL (mol/L)	2.8 ± 0.9	2.8 ± 0.7	2.4 ± 0.8

	A/A (*n* = 706)	A/C (*n* = 226)	C/C (*n* = 20)
FG (mol/L)	5.3 ± 0.9	5.2 ± 0.8	5.0 ± 0.7

	A/A (*n* = 320)	A/C (*n* = 114)	C/C (*n* = 12)
SBP (mmHg)	121.8 ± 11.7	122.9 ± 12.1	117.2 ± 13.7
DBP (mm/Hg)	69.0 ± 8.5	69.1 ± 8.2	65.3 ± 5.9

Abbreviations: DBP, diastolic blood pressure; FG, fasting glucose; HBP, high blood pressure; HDL, high‐density lipoprotein; LDL, low‐density lipoprotein; SBP, systolic blood pressure; TC, Total cholesterol; TG, Triglycerides.

* Compared with C/C, *p* < .05.

### 3.4 Expression of SCD protein was repressed by Hsa‐miR‐498

**Figure 1:**
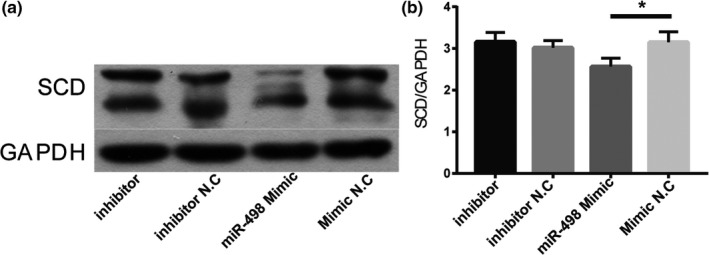
miR‐498 inhibited the expression of SCD, as detected by western blot analysis. (a, b) SCD expression in human aortic smooth muscle cells decreased following transfection with miR‐498 Mimic. *p < .05 (*n* = 3). GAPDH, glyceraldehyde 3‐phosphate dehydrogenase; miR, microRNA; N.C, negative control; SCD, stearoyl‐CoA desaturase

Rs41290540 is located in the 3′UTR of the *SCD* and may interact with miR, leading to modulation in the expression of SCD. To test that hypothesis, TargetScan Version 7.1 (http://www.targetscan.org/vert_71/) was used to predict miRNAs that might interact with rs41290540 in the 3′UTR of *SCD*. Hsa‐miR‐498 was identified using TargetScan. Next, HASMCs were transfected with the hsa‐miR‐498 Mimic, and SCD expression was evaluated using Western blot. The results are shown in Figure 1. SCD expression was significantly decreased.

### 3.5 Rs41290540 A>C variation inhibits the binding of the miR‐498

**Figure 2:**
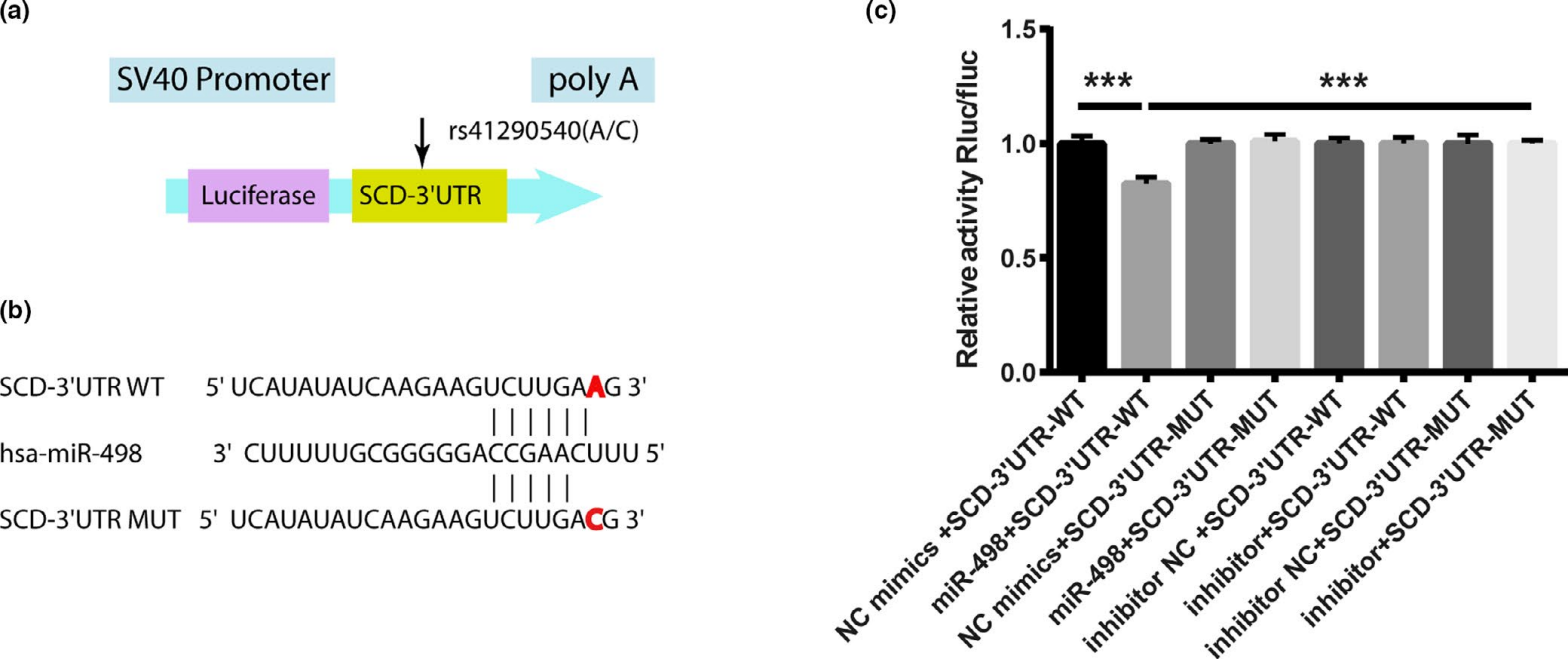
rs41290540 A>C variation in the SCD 3′UTR inhibits the binding of miR‐498. (a) Diagram of luciferase reporter constructs. (b) Diagram of the binding between the miR‐498 seed sequence and the SCD 3′UTR with the A or C allele. (c) Human embryonic kidney (HEK) 293T cells were co‐transfected with the miR‐498 Mimic/Negative.Control (N.C)/inhibitor/inhibitor N.C and luciferase reporter constructs containing the SCD 3′UTR with the A or C allele. Luciferase activity was detected using a dual luciferase assay. *p < .05 (*n* = 3). miR, microRNA; MUT, mutant; N.C, negative control; SCD, stearoyl‐CoA desaturase; UTR, untranslated region; WT, wild type

To investigate whether the SNP rs41290540 A>C variation influences miR‐498 binding, a dual‐luciferase reporter vector containing the wild‐type (A allele) and mutant (C allele) 3′UTRs and the miRNA mimic were co‐transfected into HEK293 cells. Luciferase activity was detected by a dual‐luciferase assay. The luciferase activity of the *SCD* 3′UTR was increased by the A>C variation, suggesting that the A>C variation inhibited miR‐498's binding to the target, as shown in Figure 2c.

## 4 DISCUSSION

Here, we present evidence that the rs41290540A>C variation in the 3′‐UTR of the *SCD* is related to lower TC and lower CAD's risk. Besides, we demonstrated that the rs41290540 A>C variation inhibited the binding of miR‐498 to the 3′‐UTR of *SCD* and that the expression of SCD was suppressed by miR‐498.

Atherosclerosis is a leading cause of CAD, and evidences have shown that SCD plays a beneficial role in atherosclerosis. A long‐term atherogenic diet suppresses SCD activity (Lee et al., 2017). Upregulation of SCD protects human arterial endothelial cells against lipotoxicity (Peter et al., 2008). SCD inhibition and SCD‐deficient mice develop greater inflammation and atherosclerotic lesions (Brown et al., 2010; MacDonald et al., 2009). However, in spite of plentiful in vitro and in vivo evidence, no information are available on genetic variants' role in *SCD* on CAD risk.

Genetic variation in *SCD*, including rs508384, rs10883463, rs2167444, and rs7849, is associated with enzyme activity, and rs508384 and rs7849 are also associated with obesity (Martin‐Nunez et al., 2013). Participants homozygous for the rare alleles of rs10883463, rs7849, rs2167444, and rs508384 have lower body mass index and waist circumference and ameliorated insulin sensitivity (Warensjo et al., 2007). SNPs in *SCD* alone or in cooperation with n‐3 PUFA supplementation modulated the following cardiometabolic risk factors: triglyceride (rs508384), IL6 (rs3071), CRP (rs3829160), SCD18 (18:1n‐9/18:0) indices (rs2234970), and fasting plasma glucose (rs508384) (Rudkowska et al., 2014). No study of SNP rs41290540 and its effect on CAD has been reported. A borderline association was observed between rs41290540 A>C and diabetes risk (*p* = .06); however, the same authors could not confirm these results in an independent follow‐up study (Liew et al., 2004). In this study, we found that individuals who are homozygous for the rare allele rs41290540 A>C have a lower risk of CAD. No association with hypertension and diabetes was found. However, carriers of the CC genotype had a lower TC plasma level, a predictor of CAD risk (Wilson et al., 1998); MUFAs also reduce TC (Kris‐Etherton et al., 1999); these results indicate that rs41290540 may reduce the risk of CAD by decreasing TC. The mechanism for this reduction may be that rs41290540 influences SCD to desaturate SFA, producing more MUFAs to reduce TC.

Single‐nucleotide polymorphisms located in the 3′UTR may influence gene expression by varying the interaction between the mRNA and a miRNA. Crucial recognition sites for miRNAs are nucleotides 2–7 (the seed region) at the 5′ end of the miRNA. Studies have reported that miR1925p decreases SCD expression by directly targeting the 3′UTR of *SCD* (Liu et al., 2017). Rs41290540 is located in the miR‐498 complementarity seed‐binding sequence and has the potential to affect the miRNA‐binding activity and thus affects gene transcription. MiR‐498 is involved in cellular growth (Leivonen et al., 2014). MiR‐498 is upregulated in congenital heart disease (Li et al., 2014) and stroke (Sepramaniam et al., 2014) and is a member in complex miRNA sets of novel biomarkers for acute coronary syndrome (Keller et al., 2017). In this study, the luciferase reporter assay showed that the C allele of rs41290540 attenuated the ability of miR‐498 to inhibit luciferase activity, compared to experiments utilizing the constructs harboring the A allele and miR‐498. Furthermore, our results were verified by Western blot analysis of the SCD protein levels in HASMCs. MiR‐498 overexpression resulted in a significant reduction of the SCD protein. Taken together, these experiments demonstrate that the rs41290540 A>C variation represses the binding of miR‐498, thus reducing the suppression of SCD expression.

The minor allele frequency (MAF) of rs41290540 in Asian population and our study is 0.08–0.13 (https://www.ncbi.nlm.nih.gov/snp/rs41290540) and 0.15, respectively. MAF is higher in our study; it may be related to the fact that subjects included in our study are Cantonese. Actually, MAF can vary greatly in different ethnicity {Ioannidis, Ntzani, & Trikalinos, 2004 #48}, and this may affect the prevalence and incidence of disease in different ethnic population in case of connection {Lan et al., 2007 #49}{Myles, Davison, Barrett, Stoneking, & Timpson, 2008 #50}.

There are several limitations in our study. First, we did not evaluate the expression level of SCD from different alleles in the population. In addition, this is an in vitro study. Thus, the association between SCD and rs41290540 could not be well established. Furthermore, we performed the study only in the Han Chinese population. More studies in various ethnic groups are needed to confirm the role of this polymorphism. Finally, a limitation of the present study is that the protective role of the rs41290540 A>C variation on CAD in vivo still needs to be investigated.

Taken together, our study shows that the *SCD* rs41290540 may reduce the risk of CAD via interfering with miR‐498 binding.

## CONFLICT OF INTEREST

The authors have no conflict of interest to declare.

## AUTHOR CONTRIBUTIONS

XX and KL conceived and designed the study. ZL, HM, XY, and GL performed the study; ZJL, HZ, and SW analyzed the data; LH, SL, YM, WP, JL, FY, and HT contributed samples/materials/reagents; LC and BZ wrote and drafted the manuscript.

## Data Availability

The data that support the findings of this study are available on request from the corresponding author. The data are not publicly available due to privacy or ethical restrictions.
